# Long-Term Follow-Up of T Cell Immunity Against *Orthopoxviruses* in People Living with HIV After Vaccination and Natural Monkeypox Virus Infection

**DOI:** 10.3390/vaccines13090975

**Published:** 2025-09-13

**Authors:** Monika Lindemann, Stefanie Sammet, Felix Maischack, Gabriela Graf, Peter A. Horn, Heidi Wiehler, Jessica Wunderling, Stefan Esser

**Affiliations:** 1 Institute for Transfusion Medicine, University Hospital Essen, University Duisburg-Essen, 45147 Essen, Germanypeter.horn@uk-essen.de (P.A.H.);; 2 Department of Dermatology, University Hospital Essen, University Duisburg-Essen, 45147 Essen, Germany; stefanie.sammet@uk-essen.de (S.S.); felix.maischack@uk-essen.de (F.M.); heidi.wiehler@uk-essen.de (H.W.); stefan.esser@uk-essen.de (S.E.)

**Keywords:** long-term follow-up, T cell responses, mpox, vaccination, infection, HIV, ELISpot, interferon-gamma, interleukin-2

## Abstract

Background/Objectives: After the 2022 mpox outbreak also outside Africa, risk groups including people living with HIV (PLWH) were vaccinated with the Modified Vaccinia Ankara–Bavarian Nordic vaccine (MVA-BN). Previous data on PLWH showed that two vaccinations induced specific T cell responses in 64% of the patients and natural monkeypox virus (MPXV) infection in 100%. The initial T cell response assay took place at a median of approximately 100 days post-vaccination and 300 days post-infection. Methods: This study investigates the durability of T cell immunity in PLWH by retesting patients approximately two years after initial assessment. We were able to retest 27 of 33 vaccinated patients and 7 of 10 patients after MPXV infection. T cells were stimulated with the same orthopoxvirus-derived peptide pools as in the initial study, and interferon (IFN)-γ and interleukin (IL)-2 ELISpot assays were performed. Results: The ELISpot assays showed specific T cell responses in 59% and 86% of twice vaccinated and previously infected patients, respectively. Paired analysis revealed no significant differences between previous and current data (short- and long-term follow-up), with IL-2 ELISpot results showing positive correlations at both time points (*r* = 0.67, *p* = 0.0001). Long-term IFN-γ responses after MPXV infection were 4.3 times higher (*p* < 0.01), and IL-2 responses were 2.9 times higher (*p* = 0.05) than after vaccination. Conclusions: Our data indicates that T cell responses to *Orthopoxviruses* remain overall stable for 2–3 years in PLWH, with long-term immunity being stronger after natural MPXV infection than after two vaccinations.

## 1. Introduction

In May 2022, an outbreak of mpox (formerly called “monkeypox”) rapidly spread across Europe, the Americas, and subsequently all six WHO regions [1]. The global outbreak has affected primarily gay, bisexual, and other men who have sex with men and has spread from person-to-person through sexual networks [1]. The illness presents with symptoms similar to smallpox, though they are generally less severe except in people living with HIV (PLWH) and advanced immunodeficiency [1]. Mpox has caused more than 142,000 cases in 130 total countries so far [2,3]. Mpox, formerly recognized as a viral zoonosis, caused by an enveloped double-stranded DNA virus of the genus *Orthopoxvirus* (family *Poxviridae*), was first scientifically recognized in a human in the Democratic Republic of Congo in 1970 and has been endemic in Central, Eastern, and West Africa, where the clades I and II of the virus could be detected [1]. In 2022–2023, the global outbreak of mpox was caused by the subclade IIb of the virus [1,2]. Mpox still remains a threat today, and there are outbreaks, especially of clade I, in Central and Eastern Africa [1,2]. Moreover, clade II mpox cases continue to spread at low levels in many countries around the world [2].

Since June 2022, at-risk populations have been vaccinated with vaccines also effective against smallpox, such as the live, attenuated vaccinia virus strain Ankara (Modified Vaccinia Ankara–Bavarian Nordic (MVA-BN), marketed as Jynneos in the USA and Imvanex in Europe) [4]. MVA-BN is FDA-approved for mpox prevention and is considered safe for immunocompromised individuals, including PLWH [5,6,7,8]. The T cell response against Monkeypox virus (MPXV) primarily involves the activation and proliferation of antigen specific T cells, which can be divided into CD4+ helper T cells and CD8+ cytotoxic T cells. Studies indicate that during natural MPXV infection, individuals exhibit substantial expansion of both CD4+ and CD8+ T cells, which express markers indicative of activation and effector function, especially in patients demonstrating a strong response characterized by a Th1-type immune profile [9]. In viral infections such as mpox, different T cell subsets secrete specific cytokines as part of the immune response: IFN-γ is mainly secreted by CD4+ Th1 cells and cytotoxic T cells, and IL-2 by CD4+ Th1 cells [10]. After vaccination with MVA-BN, the high degree of antigenic similarity between vaccinia and MPXV leads to durable, cross-reactive T cell and B-cell immunity, which underpins the protective efficacy of smallpox vaccines against MPXV infection [11].

Our previous data indicated that after two vaccinations, 64% of the patients showed specific IFN-γ and/or IL-2 responses, and after MPXV infection all patients responded [12]. In patients after infection, specific IFN-γ secretion was significantly (*p* < 0.001) higher than after vaccination.

The current study addresses the question of how stable T cell immunity to *Orthopoxviruses* was in PLWH. T cell responses were measured by counting the number of cytokine-secreting T cells, using the highly sensitive ELISpot assay. The initial examination of T cell responses [12] took place at a median of 115 days after completion of the 2nd dose of vaccination and 314 days after mpox diagnosis (by a physician and/or PCR test), referred to as short-term immunity. We have now followed up with the patients two years later (long-term immunity) and were able to retest 27 of 33 vaccinated patients and 7 of 10 patients after MPXV infection. We analyzed whether the strength of immunity had changed, whether there was a difference between patients after vaccination and infection, whether there was a correlation between short- and long-term immunity, and whether the number of T cells or age correlated with the strength or course of T cell function.

## 2. Subjects and Methods

### 2.1. Subjects

The patient cohort included 34 male PLWH with a median age of 46 years (range 25–65) and a median CD4+ T cell count of 817/μL (range 286–1590). Twenty-seven patients were tested after vaccination with MVA-BN smallpox/mpox prophylactic vaccine (Imvanex, Bavarian Nordic A/S, Kvistgaard, Denmark) and seven patients after MPXV infection (Table 1).

With the exception of two patients, all vaccinated subjects had received a 2-dose regimen with the MVA-BN vaccine, 0.5 mL each, at intervals of 28–227 days (median 119 days). The other two patients had evidence of vaccination against smallpox in early childhood and had recently received a 1st booster vaccination with MVA-BN.

It is noteworthy that none of the subjects infected with mpox had been vaccinated prior to infection (neither recently with MVA-BN nor in childhood with another smallpox vaccine). However, one PLWH was vaccinated with MVA-BN four days after a very likely exposure to mpox and was re-exposed one day after the mpox vaccination. Nine days after the first exposure, he developed the first symptoms of mpox (skin lesions). This subject did not receive any further vaccination. None of the other PLWH infected with mpox have received the MVA-BN vaccine to date.

None of the patients had progressed to AIDS within six months prior to testing or thereafter. All PLWH had been receiving antiretroviral treatment (ART) for at least 6 months at the time of vaccination, and ART was continued during the current follow-up period. Apart from a few sporadic, transient increases in plasma viral load (“blips”), all ART-treated PLWH remained virologically suppressed throughout the observation period.

Cellular immune responses were analyzed at a median of 820 days after the 2nd dose of vaccination or at a median of 974 days after MPXV infection.

This study was conducted according to the guidelines of the Declaration of Helsinki and approved by the Ethics Committee of the University Hospital Essen, Germany (SCABIO-HIV). Informed consent was obtained from all subjects involved in this study.

### 2.2. ELISpot Assay

To measure orthopoxvirus-specific cellular immunity, in house ELISpot assays for IFN-γ and IL-2 were performed as described previously [12]. In brief, we stimulated 300,000 peripheral blood mononuclear cells (PBMCs) with a mix of 127 peptides covering selected proteins of the MPXV, smallpox (variola) virus, and vaccinia virus (PepMix Pan-*Poxviridae* Select, jpt, Berlin, Germany). According to information by jpt, 91% of the epitopes covered by the peptide pool (115/127) are shared between vaccinia virus (strain Ankara) and MPXV (strain Zaire-96-I-16) (https://media.jpt.com/Media/Documents/Annotations/Annotations_PM-Pan-PXVselect-1.pdf, accessed on 17 July 2025). The lyophilized peptide pool (25 μg per peptide) was resuspended in 40 μL of dimethyl sulfoxide (DMSO, SERVA, Heidelberg, Germany). It was then diluted to a final concentration of 1 μg/mL per peptide with AIM V™ medium (Thermo Fisher Scientific, Frankfurt am Main, Germany), which was also used (without peptide stimulation) for the negative controls. Thus, the DMSO concentration in the cell cultures was 0.16% (*v*/*v*) which is considered as non-toxic (https://media.jpt.com/Media/Documents/Protocol/Protocol_PepMix.pdf, accessed on 17 July 2025). All assays included negative and positive controls, i.e., cultures left unstimulated or stimulated with the mitogen phytohemagglutinin (1 μg/mL). Cell cultures were performed for 19–24 h, and cytokine spots were detected by an ELISpot plate reader (AID Fluorospot, Autoimmun Diagnostika GmbH, Strassberg, Germany). Mean values of duplicate cell cultures were considered. As a result, we determined orthopoxvirus-specific responses as spot increments, defined as stimulated minus unstimulated values, i.e., the increment in Spot-Forming Units (SFU). The negative controls reached a median value of 0 IFN-γ and 1 IL-2 spots, the positive controls 686 IFN-γ and >800 IL-2 spots, respectively.

Stimulated spot numbers >3-fold higher than negative (unstimulated) controls combined with an increment value of at least 4 were considered positive, based on previous studies [12,13]. In a study by Sammet et al. [12], we used exactly the same ELISpot assay as in the current study. In healthy volunteers, the negative controls reached a median value of 0 IFN-γ spots and a mean value of 0.3 (range 0–1.5). For IL-2 spots, the median was 0 and the mean 0.5 (range 0–2). For IFN-γ the mean negative control plus 3-fold standard deviation was 1.9 spots, for the IL-2 it was 2.5 spots. In the study by Schwarzkopf et al. [13], very similar assay conditions were used. However, 250,000 instead of 300,000 PBMCs per culture were used in the current study, and thus the cut-off in the current study was slightly adapted (an increment of 4 spots instead of 3).

### 2.3. Statistical Analysis

Statistical analysis was performed with GraphPad Prism 8.4.2.679 (San Diego, CA, USA). Short- and long-term data on cellular immunity were compared by a Wilcoxon matched pairs test, data in vaccinated and infected patients by a Mann–Whitney test. For the analysis of numerical variables, we used Spearman correlation analysis (basic Spearman rank correlation). Two-sided *p* values < 0.05 were considered significant.

## 3. Results

### 3.1. Comparison of Short- and Long-Term Cellular Immunity Against Orthopoxviruses

**Figure 1:**
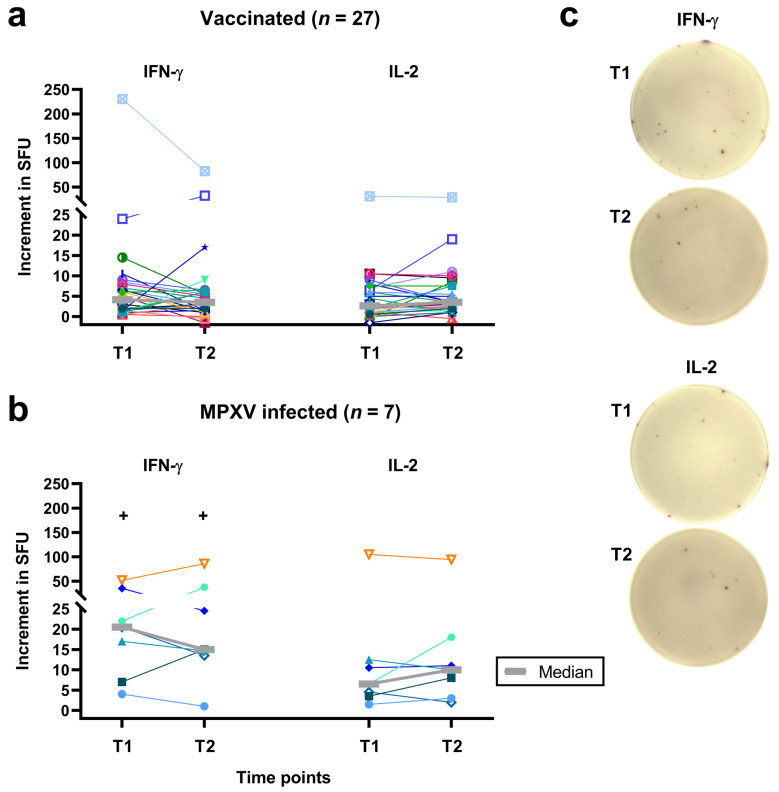
Follow-up of individual results of orthopoxvirus-specific IFN-γ and IL-2 ELISpot assays. Panel (**a**) shows data on 27 people living with HIV after two vaccinations against smallpox and panel (**b**) on 7 people living with HIV after monkeypox virus (MPXV) infection. Panel (**c**) shows images of orthopoxvirus-specific IFN-γ and IL-2 ELISpot assays in a vaccinated subject. Median values are displayed as gray, bold lines. The increment in Spot-Forming Units (SFU) indicates that orthopoxvirus-specific values minus negative controls are displayed. T1 means short-term data, as determined in a previous study [12] and T2 long-term data, as measured in the current study, which was two years after T1 (median 820 days after 2nd dose of vaccination and 974 days after diagnosis of MPXV infection). Data on each individual are shown by a unique symbol/color, data on the subject who was vaccinated after exposure to mpox are shown with turquoise triangles. Data at T1 and T2 were compared by a Wilcoxon matched pairs test (none of the differences was statistically significant), data in vaccinated and infected patients by a Mann–Whitney test (+*p* < 0.01).

We compared the results of an orthopoxvirus-specific ELISpot assay in 34 PLWH at two timepoints, T1 (median 115 days after the 2nd dose of vaccination and 314 days after mpox diagnosis) [12] and T2 (median 820 days after vaccination and 974 days after MPXV infection, i.e., two years later). Neither 27 patients following vaccination nor 7 patients following MPXV infection showed a significant difference in cellular immunity between T1 and T2 (Figure 1a–c). However, IFN-γ responses decreased slightly from T1 to T2, while IL-2 responses increased slightly. In the long-term follow-up (T2), median IFN-γ responses in patients after MPXV infection were 4.3 times higher than after vaccination (*p* < 0.01). A similar trend was observed for IL-2 responses, which were 2.9 times higher (*p* = 0.05). For comparison, at T1 IFN-γ responses were 4.7-fold higher and IL-2 responses 2.7-fold higher after infection vs. vaccination [12]. After vaccination, orthopoxvirus-specific T cells reached a median frequency of 3.5 IFN-γ and 3.5 IL-2 secreting cells per 300,000 PBMCs (0.001%). After MPXV infection, specific T cells reached a median frequency of 15 IFN-γ and 10 IL-2 secreting cells per 300,000 PBMCs (0.005% and 0.003%, respectively).

Considering individual courses, ten of the 27 vaccinated patients showed an increase in the IFN-γ response from T1 to T2, one a stable response and 16 a decreased response. Fourteen of the vaccinees displayed an increase in the IL-2 response, 3 a stable response and 10 a decreased response (Figure 1a,c). Moreover, 3 of the 7 mpox infected patients showed an increase in the IFN-γ response and 4 a decreased response. Four of the patients with prior infection displayed an increase in the IL-2 response and 3 a decreased response (Figure 1b). ELISpot responses for the person who was vaccinated after exposure to MPXV (represented by turquoise triangles) were similar to median responses.

**Figure 2:**
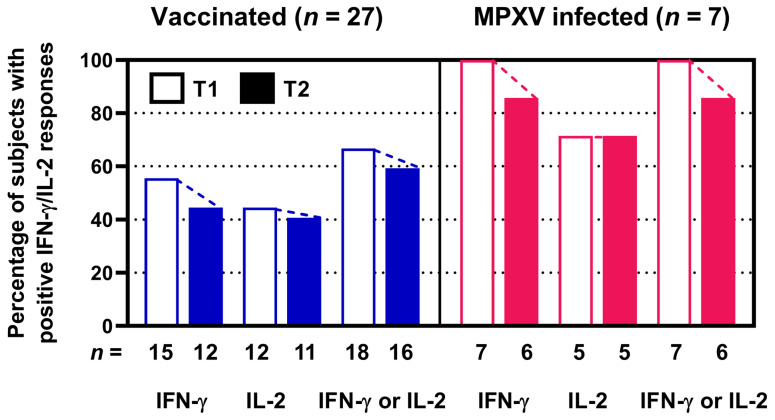
Percentage and absolute numbers of positive orthopoxvirus-specific ELISpot assay results in 27 people living with HIV after two vaccinations against smallpox ((**left**) panel) and in 7 people living with HIV after monkeypox virus (MPXV) infection ((**right**) panel). Stimulated spot numbers >3-fold higher than negative (unstimulated) controls combined with an increment value of at least 4 were considered positive. Open bars indicate short-term data determined in a previous study (T1) [12]. Filled bars show long-term data at T2, as measured in the current study, which was two years after T1. *n* denotes the number of subjects. We assessed IFN-γ and IL-2 responses separately and combinedly (IFN-γ or IL-2).

As a second step, we analyzed the rate of positive ELISpot responses at T1 and T2 (Figure 2). We observed that after vaccination the number of positive IFN-γ responses declined from 15 to 12 out of 27 and of positive IL-2 responses from 12 to 11 out of 27. After infection positive IFN-γ responses declined from 7 to 6 out of 7 and positive IL-2 responses remained constant at 5 out of 7. When analyzing IFN-γ and IL-2 responses combinedly, positive responses after vaccination declined from 18 to 16 out of 27 and after infection from 7 to 6 out of 7, i.e., from 67 to 59% and from 100 to 86%, respectively. Thus, a minor decline in the positivity rate could be found.

### 3.2. Correlation Between Short- and Long-Term Immunity Against Orthopoxviruses

**Figure 3:**
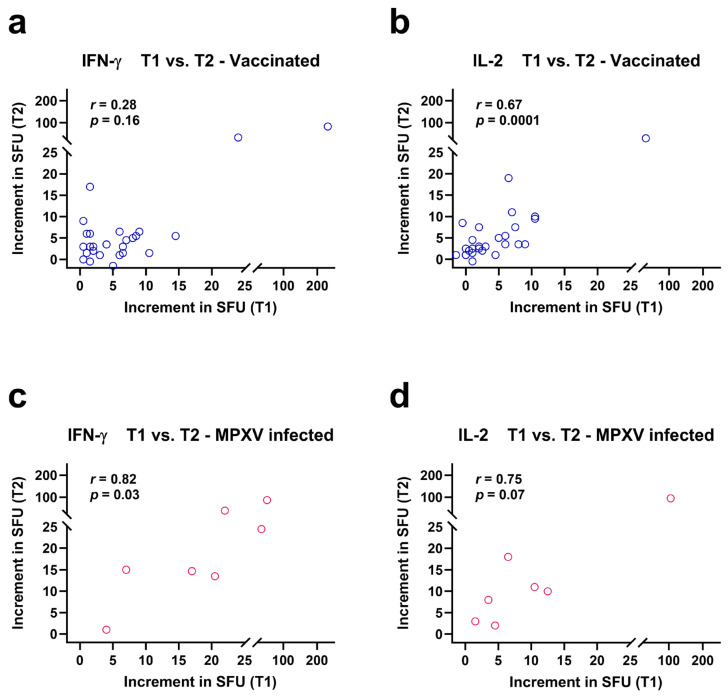
Spearman correlation analysis of orthopoxvirus-specific IFN-γ and IL-2 ELISpot results at two time points, T1 and T2. Data were analyzed separately in 27 people living with HIV after two vaccinations against smallpox (**a**,**b**) and in 7 people living with HIV after monkeypox virus (MPXV) infection (**c**,**d**). The increment in Spot-Forming Units (SFU) indicates that orthopoxvirus-specific values minus negative controls are displayed. Short-term data (T1) were determined in a previous study [12] and long-term data (T2) were measured in the current study. Both time points were approximately two years apart from each other.

ELISpot responses against *Orthopoxviruses* were compared at the time points T1 and T2, which were approximately two years apart (Figure 3). Spearman correlation analysis indicated that IFN-γ responses at T1 and T2 tended to correlated positively in vaccinated patients (*r* = 0.28, *p* = 0.16) (Figure 3a) and IL-2 responses showed significant positive correlation (*r* = 0.67, *p* = 0.0001) (Figure 3b). Correlation coefficients were even higher in patients after MPXV infection (IFN-γ: *r* = 0.82, *p* = 0.03; IL-2: *r* = 0.75, *p* = 0.07) (Figure 3c,d).

### 3.3. Comparison of Short- and Long-Term Data on T cell Subpopulations

**Figure 4:**
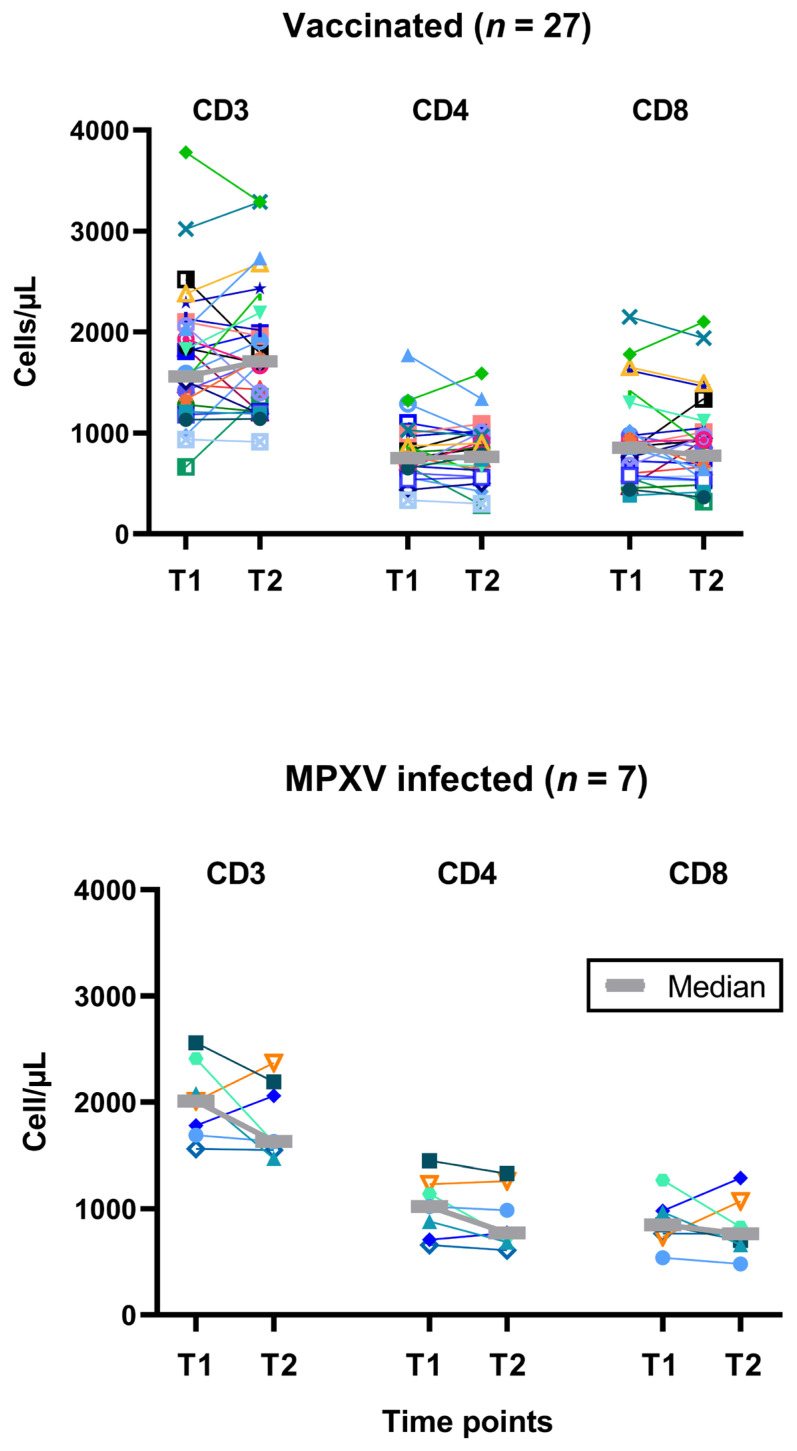
Follow-up of individual results of T cell subpopulations. The upper panel shows data on 27 people living with HIV after two vaccinations against smallpox and the lower panel on 7 people living with HIV after monkeypox virus (MPXV) infection. We used the same symbols as in Figure 1. T1 means short-term data, as determined in a previous study [12] and T2 long-term data, as measured in the current study, which was two years after T1 (median 820 days after 2nd dose of vaccination and 974 days after diagnosis of infection). Data on the subject who was vaccinated after exposure to mpox is shown with turquoise triangles.

In parallel to the T cell immunity against *Orthopoxviruses*, T cell subpopulations were determined in vaccinated and mpox-infected PLWH. CD3+, CD4+ and CD8+ T cells did not change significantly during the 2-years follow-up (Figure 4). However, CD4+ T cell counts tended to be higher in infected vs. vaccinated subjects at T1 (median values: 1020 vs. 753/µL, *p* = 0.09). At T2, both groups displayed similar values for CD4+ T cells.

### 3.4. Correlation Analysis of the Number of T Cells or Age with the Strength or Course of T Cell Function

**Figure 5:**
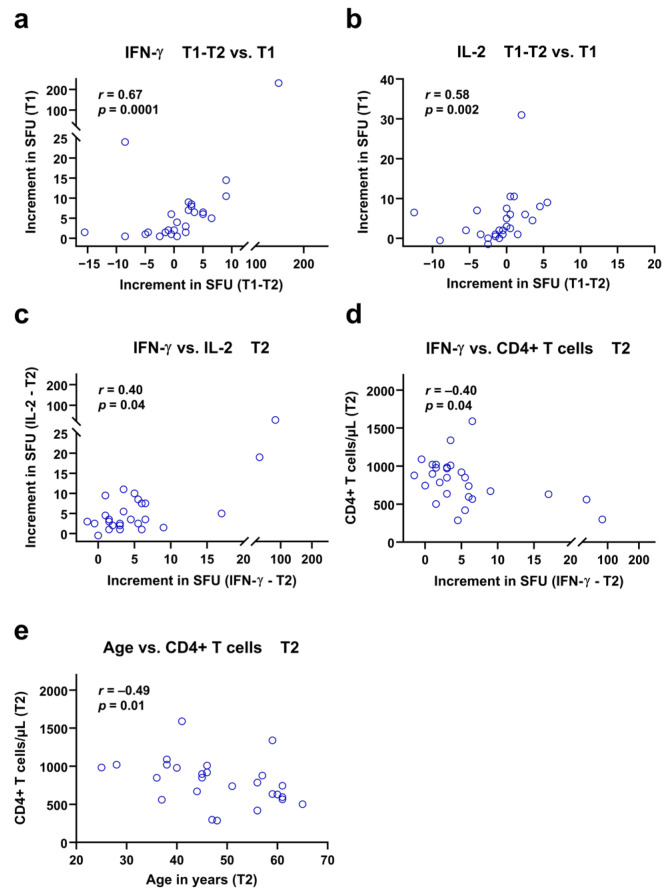
Spearman correlation analyses of ELISpot responses against *Orthopoxviruses* and clinical or in vitro parameters in 27 vaccinated people living with HIV. The increment in Spot-Forming Units (SFU) indicates that orthopoxvirus-specific values minus negative controls are displayed. The analysis considers long-term data of the current study (T2), short-term data (T1) of a previous study [12] and the difference between short- and long-term data (T1-T2). The time points T1 and T2 were approximately two years apart from each other. Patients who showed high specific IFN-γ and IL-2 responses early after vaccination (T1) also showed a greater decline in responses during the follow-up period (T1-T2), as shown in panels (**a**,**b**). In addition, the responses to the IFN-γ and IL-2 ELISpot assays at T2 showed a moderate but significant positive correlation (**c**). Finally, panels (**d**,**e**) indicate negative correlations of IFN-γ responses and age with CD4+ T cell counts at T2.

Using Spearman analysis, we tested if numbers of T cells and patient age as well as previous cellular immune function (at T1) correlated with ELISpot responses of the current study (T2). Moreover, the difference in ELISpot responses between short- and long-term follow up (T1-T2) was taken into consideration. These analyses were performed separately for patients after vaccination and infection. While no correlation reached statistical significance in mpox-infected PLWH, several correlations were significant in vaccinated subjects (Figure 5). The highest correlation coefficient was observed for the change in IFN-γ ELISpot responses (T1-T2) and IFN-γ ELISpot results at T1 (*r* = 0.67, *p* = 0.0001) (Figure 5a). Thus, patients with a stronger initial response to vaccination also showed a more pronounced decline of responses. A similar finding was observed for the IL-2 ELISpot, where also the initial vaccination response at T1 correlated positively with the decline of responses (*r* = 0.58, *p* = 0.002) (Figure 5b). Moreover, responses to the IFN-γ ELISpot at T2 correlated positively with responses to the IL-2 ELISpot at T2 (*r* = 0.40, *p* = 0.04) (Figure 5c). After Bonferroni correction, however, this last correlation no longer remained significant, similar to inverse correlations of absolute CD4 T cell counts with IFN-γ ELISpot results and age at T2 (Figure 5d,e).

## 4. Discussion

In 34 PLWH, the strength of T cell immunity against *Orthopoxviruses* did not change significantly during a two-year follow-up period, neither after vaccination with MVA-BN nor after MPXV infection. Similarly to our previous data on PLWH [12], in the current study specific IFN-γ secretion was 4.3 times higher after MPXV infection than after vaccination and IL-2 secretion was 2.9 times higher. Thus, infection with mpox in PLWH still induced stronger T cell immunity in the long-term, i.e., after 2–3 years, than two vaccinations, which has not yet been published. Similarly to the current data, Agrati et al. found that almost all patients with MPXV infection (15 of 16) developed an orthopoxvirus-specific Th1 response, which was analyzed within 10–12 days after infection [14]. Of note, vaccinated PLWH received a vaccine containing vaccinia virus antigens, whereas the infected group had contact with MPXV. Ninety-one percent of the epitopes covered by the peptide pool (115/127) we used in our ELISpot assay are shared between vaccinia virus (strain Ankara) and MPXV. Therefore, the 2.9 to 4.3 times higher ELISpot response in mpox infected vs. vaccinated PLWH cannot be attributed solely due to a contact with various virus antigens. The immune system developed a stronger and presumably also broader immunity after natural infection with mpox than after vaccination.

Our finding of a rather stable T cell immunity after vaccination with MVA-BN and after MPXV infection is in line with clinical data pointing to long-term (partial) protection after smallpox vaccination. Studies performed prior to the outbreak of mpox in 2022 have found that adults who received smallpox vaccination during childhood experienced either milder symptoms or remained asymptomatic following MPXV infection [15,16,17,18]. Additionally, evidence from an mpox outbreak in Zaire demonstrated that smallpox vaccination provided substantial clinical protection during such outbreaks [19]. In research conducted between 1980 and 1984, investigators in Zaire examined 2510 individuals who had been in contact with 214 mpox patients. The attack rate among unvaccinated contacts was 7.2%, compared to just 0.9% among those with a history of smallpox vaccination. But these previous studies [15,16,17,18,19] did not report on cohorts of PLWH. Moreover, it was reported in 2023 that healthy controls historically vaccinated against smallpox can maintain a residual T cell response for decades after vaccination [20].

Data on specific T cell immunity after MPXV infection during the current mpox outbreak are scarce, especially in PLWH. Agrati et al. [14] and Sistere-Oro et al. [21] assessed specific T cell responses by ELISpot until day 10–12 or day 20 after MPXV infection, respectively, and Mazzotta et al. [22] until month 1 after the 2nd dose of vaccination, when T cell responses increased significantly as compared to baseline. Moreover, we previously published T cell data approximately 100 days post-vaccination and 300 days post-infection [12]. In these four studies, T cell responses were consistently observed in a subgroup of PLWH. In line with the in vitro data, current observational studies including PLWH indicate that natural immunity and vaccine-induced immunity are not fully protective against MPXV infection [23,24]. A recent multicenter, observational study enrolling men who have sex with men and transgender people aged 18 years or older with changing sexual partners in Germany [24] showed that the effectiveness of one vaccination dose was 35% in PLWH and 84% in people without HIV. Among the 211 individuals who received two doses of MVA-BN vaccination (including PLWH), no mpox cases occurred. Of note, the median follow-up was 55 days after the 1st dose of vaccination, and 14 days after the 2nd dose of vaccination [24]. In addition, a large case–control study including PLWH and individuals receiving pre-exposure prophylaxis against HIV infection found that the effectiveness of one vaccination with MVA-BN was 36% and the effectiveness of two vaccinations was 66% [25]. Supporting the assumption of partial effectiveness of vaccination with MVA-BN in PLWH, vaccinated vs. unvaccinated individuals showed markedly reduced symptom severity, with less disseminated mpox lesions and fewer systemic symptoms including fever [24].

Before Bonferroni correction, a weak inverse correlation (*r* = −0.46, *p* = 0.02) between the number of CD4+ T cells and IFN-γ ELISpot responses was determined in vaccinated subjects at T2. The result was unexpected. It also does not fit with the relative CD4+ T cells, for which no correlation with the ELISpot responses was found (*r* = −0.17, *p* = 0.4). Furthermore, the ELISpot responses did not correlate with the CD8+ T cells (absolute values: *r* = −0.18, *p* = 0.4; relative values: *r* = 0.25, *p* = 0.2). Most likely, the statistical significance occurred by chance. However, we did not want to leave this result unaddressed. We had expected a positive correlation between CD4+ T cells and IFN-γ ELISpot reactions, as recently described after SARS-CoV-2 vaccination [26]. A research group at the US NIH found that after the first vaccination, 7% of individuals with CD4 counts < 200/µL had a detectable T cell-mediated immune response, as measured by an ELISA to detect interferon-gamma release, compared to 35% of individuals with CD4 counts > 500/µL. Our result may be due to the fact that only 32 of 34 PLWH had CD4+ T cell counts > 400/µL, which makes the correlation analysis less meaningful.

In this context, some limitations of our study must be considered. The study cohort is relatively small. Since only a few patients had low CD4+ T cell counts, the data on correlation of T cell subpopulations and ELISpot responses should be viewed with caution and confirmed. In addition, the ELISpot assay uses PBMCs, so the responses mediated by CD4+ or CD8+ T cells cannot be evaluated separately, and the polyfunctionality of the response cannot be assessed. Finally, we used only a single method to test T cell immunity (ELISpot assay) and did not consider B-cell immunity. However, a strength of this study is that it describes long-term data (tracking T cell immunity over a period of two years) in a very specific cohort, including people with HIV infection and MPXV infection. The sequential analysis was performed using exactly the same method and by the same technicians. The patients were also treated by the same staff as in our previous study. This makes it easy to compare these paired data.

It is challenging to determine an optimal threshold value for positive T cell responses that also indicate protective immunity. MPXV is not only controlled by T cells but also by neutralizing antibodies [20,27,28,29,30], which complicates the cut-off definition. Nevertheless, T cell immunity is considered as decisive factor for long-term protection, especially against severe diseases caused by viruses such as SARS-CoV-2 [31]. Furthermore, in MPXV infection, T cells are likely to provide an important contribution to overall immunity [32]. The threshold value for positive responses that we applied in the current study was already validated in an earlier study [12], among other things by comparison with various negative controls. Using our cut-off definition, vaccination with MVA-BN resulted in positive responses in 64% of PLWH at a median of 115 days after the 2nd dose of vaccination and in 59% of patients a median of 820 days after the 2nd dose of vaccination. Overall, these values are in the same range as the protection rates reported (66% after two vaccinations) [25].

## 5. Conclusions

The results of our small-scale study are good news for vaccinated PLWH. They indicate that T cell immunity against *Orthopoxviruses* decreases minimally and insignificantly within two years after two vaccinations with MVA-BN. Nevertheless, breakthrough infections despite vaccination occur [27,28,29,30]. They are, however, usually less severe than infections without prior vaccination. This reflects the public health principle of a layered defense, where multiple interventions work together to achieve optimal protection against infection.

## Figures and Tables

**Table 1:** Characteristics of 34 people living with HIV tested for cellular immunity against *Orthopoxviruses*.

Variable ^1^	Vaccinated (*n* = 27)	MPXV Infected (*n* = 7)	*p* Value
Age (years)	46 (25–65)	46 (31–53)	
Interval 2nd vaccination or infection—testing (days) ^2^	820 (700–910)	974 (924–1026)	<0.0001
Absolute cell counts (cells/μL)			
CD3+	1810 (664–3780)	1630 (1470–2370)	
CD4+	848 (286–1590)	772 (610–1330)	
CD8+	877 (321–2100)	763 (480–1290)	
Relative cell counts [%]			
CD3+	74.0 (53.8–81.6)	73.3 (70.3–86.4)	
CD4+	30.7 (20.2–40.2)	37.5 (26.2–48.8)	
CD8+	38.8 (20.1–52.4)	36.4 (23.0–43.9)	
CD4/CD8 ratio	1.0 (0.4–2.2)	1.0 (0.6–2.1)	

^1^ Data are given as median (range). ^2^ The interval between the 2nd dose of vaccination or monkeypox virus (MPXV) infection and testing was named T2, in contrast to the interval assessed previously which was named T1 [12]. Characteristics of the two patient groups were compared by a Mann–Whitney test. Significant differences were found only for the interval between vaccination/infection and testing, as indicated.

## Data Availability

The data presented in this study are available on request from the corresponding author. The data are not publicly available due to privacy restrictions.

## References

[1] WHO Mpox Key Facts. https://www.who.int/news-room/fact-sheets/detail/mpox.

[2] CDC Mpox in the United States and Around the World: Current Situation. https://www.cdc.gov/mpox/situation-summary/.

[3] WHO Mpox: Multi-country External Situation Report No. 53 Published 29 May 2025. https://cdn.who.int/media/docs/default-source/documents/emergencies/multi-country-outbreak-of-mpox--external-situation-report--53.pdf.

[4] EMA Imvanex: EPAR—Product Information. https://www.ema.europa.eu/en/documents/product-information/imvanex-epar-product-information_en.pdf.

[5] Earl P.L., Americo J.L., Wyatt L.S., Eller L.A., Whitbeck J.C., Cohen G.H., Eisenberg R.J., Hartmann C.J., Jackson D.L., Kulesh D.A. (2004). Immunogenicity of a highly attenuated MVA smallpox vaccine and protection against monkeypox. Nature.

[6] Aden D., Zaheer S., Kumar R., Ranga S. (2023). Monkeypox (Mpox) outbreak during COVID-19 pandemic-Past and the future. J. Med. Virol..

[7] Overton E.T., Lawrence S.J., Stapleton J.T., Weidenthaler H., Schmidt D., Koenen B., Silbernagl G., Nopora K., Chaplin P. (2020). A randomized phase II trial to compare safety and immunogenicity of the MVA-BN smallpox vaccine at various doses in adults with a history of AIDS. Vaccine.

[8] Walsh S.R., Wilck M.B., Dominguez D.J., Zablowsky E., Bajimaya S., Gagne L.S., Verrill K.A., Kleinjan J.A., Patel A., Zhang Y. (2013). Safety and immunogenicity of modified vaccinia Ankara in hematopoietic stem cell transplant recipients: A randomized, controlled trial. J. Infect. Dis..

[9] Cohn H., Bloom N., Cai G.Y., Clark J.J., Tarke A., Bermudez-Gonzalez M.C., Altman D.R., Lugo L.A., Lobo F.P., Marquez S. (2023). Mpox vaccine and infection-driven human immune signatures: An immunological analysis of an observational study. Lancet Infect. Dis..

[10] Murphy K.M., Weaver C., Murphy K.M., Weaver C. (2023). The Adaptive Immune Response—T Cell Mediated Immunity. Janeway’s Immunobiology.

[11] Crandell J., Monteiro V.S., Pischel L., Fang Z., Conde L., Zhong Y., Lawres L., de Asis G.M., Maciel G., Zaleski A. (2025). The impact of orthopoxvirus vaccination and Mpox infection on cross-protective immunity: A multicohort observational study. Lancet Microbe.

[12] Sammet S., Koldehoff M., Schenk-Westkamp P., Horn P.A., Esser S., Lindemann M. (2024). T Cell Responses against Orthopoxviruses in HIV-Positive Patients. Vaccines.

[13] Schwarzkopf S., Krawczyk A., Knop D., Klump H., Heinold A., Heinemann F.M., Thummler L., Temme C., Breyer M., Witzke O. (2021). Cellular Immunity in COVID-19 Convalescents with PCR-Confirmed Infection but with Undetectable SARS-CoV-2-Specific IgG. Emerg. Infect. Dis..

[14] Agrati C., Cossarizza A., Mazzotta V., Grassi G., Casetti R., De Biasi S., Pinnetti C., Gili S., Mondi A., Cristofanelli F. (2023). Immunological signature in human cases of monkeypox infection in 2022 outbreak: An observational study. Lancet Infect. Dis..

[15] Dubois M.E., Slifka M.K. (2008). Retrospective analysis of monkeypox infection. Emerg. Infect. Dis..

[16] Sejvar J.J., Chowdary Y., Schomogyi M., Stevens J., Patel J., Karem K., Fischer M., Kuehnert M.J., Zaki S.R., Paddock C.D. (2004). Human monkeypox infection: A family cluster in the midwestern United States. J. Infect. Dis..

[17] Hammarlund E., Lewis M.W., Carter S.V., Amanna I., Hansen S.G., Strelow L.I., Wong S.W., Yoshihara P., Hanifin J.M., Slifka M.K. (2005). Multiple diagnostic techniques identify previously vaccinated individuals with protective immunity against monkeypox. Nat. Med..

[18] Karem K.L., Reynolds M., Hughes C., Braden Z., Nigam P., Crotty S., Glidewell J., Ahmed R., Amara R., Damon I.K. (2007). Monkeypox-induced immunity and failure of childhood smallpox vaccination to provide complete protection. Clin. Vaccine Immunol..

[19] Ligon B.L. (2004). Monkeypox: A review of the history and emergence in the Western hemisphere. Semin. Pediatr. Infect. Dis..

[20] Sammartino J.C., Cassaniti I., Ferrari A., Piralla A., Bergami F., Arena F.A., Paolucci S., Rovida F., Lilleri D., Percivalle E. (2023). Characterization of immune response against monkeypox virus in cohorts of infected patients, historic and newly vaccinated subjects. J. Med. Virol..

[21] Sistere-Oro M., Du J., Wortmann D.D.J., Filippi M.D., Canas-Ruano E., Arrieta-Aldea I., Marcos-Blanco A., Castells X., Grau S., Garcia-Giralt N. (2024). Pan-pox-specific T-cell responses in HIV-1-infected individuals after JYNNEOS vaccination. J. Med. Virol..

[22] Mazzotta V., Lepri A.C., Matusali G., Cimini E., Piselli P., Aguglia C., Lanini S., Colavita F., Notari S., Oliva A. (2024). Immunogenicity and reactogenicity of modified vaccinia Ankara pre-exposure vaccination against mpox according to previous smallpox vaccine exposure and HIV infection: Prospective cohort study. eClinicalMedicine.

[23] Hazra A., Zucker J., Bell E., Flores J., Gordon L., Mitja O., Suner C., Lemaignen A., Jamard S., Nozza S. (2023). Mpox in people with past infection or a complete vaccination course: A global case series. Lancet Infect. Dis..

[24] Hillus D., Le N.H., Tober-Lau P., Fietz A.K., Hoffmann C., Stegherr R., Huang L., Baumgarten A., Voit F., Bickel M. (2025). Safety and effectiveness of MVA-BN vaccination against mpox in at-risk individuals in Germany (SEMVAc and TEMVAc): A combined prospective and retrospective cohort study. Lancet Infect. Dis..

[25] Deputy N.P., Deckert J., Chard A.N., Sandberg N., Moulia D.L., Barkley E., Dalton A.F., Sweet C., Cohn A.C., Little D.R. (2023). Vaccine Effectiveness of JYNNEOS against Mpox Disease in the United States. N. Engl. J. Med..

[26] Antinori A., Cicalini S., Meschi S., Bordoni V., Lorenzini P., Vergori A., Lanini S., De Pascale L., Matusali G., Mariotti D. (2022). Humoral and Cellular Immune Response Elicited by mRNA Vaccination Against Severe Acute Respiratory Syndrome Coronavirus 2 (SARS-CoV-2) in People Living with Human Immunodeficiency Virus Receiving Antiretroviral Therapy Based on Current CD4 T-Lymphocyte Count. Clin. Infect. Dis..

[27] Zeggagh J., Ferraris O., Salmona M., Tarantola A., Molina J.M., Delaugerre C. (2023). Second clinical episode of hMPX virus in a man having sex with men. Lancet.

[28] Taha A.M., Rodriguez-Morales A.J., Sah R. (2023). Mpox breakthrough infections: Concerns and actions. Lancet Infect. Dis..

[29] Weerasinghe M.N., Ooi C., Kotsiou G., Cornelisse V.J., Painter A. (2023). Breakthrough mpox despite two-dose vaccination. Med. J. Aust..

[30] Mohapatra R.K., Mishra S., Rabaan A.A., Mohanty A., Padhi B.K., Sah R. (2023). Monkeypox breakthrough infections and side-effects: Clarion call for nex-gen novel vaccine. New Microbes New Infect..

[31] Florian D.M., Bauer M., Popovitsch A., Fae I., Springer D.N., Graninger M., Traugott M., Weseslindtner L., Aberle S.W., Fischer G. (2025). Enhanced and long-lasting SARS-CoV-2 immune memory in individuals with common cold coronavirus cross-reactive T cell immunity. Front. Immunol..

[32] Grifoni A., Zhang Y., Tarke A., Sidney J., Rubiro P., Reina-Campos M., Filaci G., Dan J.M., Scheuermann R.H., Sette A. (2022). Defining antigen targets to dissect vaccinia virus and monkeypox virus-specific T cell responses in humans. Cell Host Microbe.

